# A genome-wide screen for modifiers of transgene variegation identifies genes with critical roles in development

**DOI:** 10.1186/gb-2008-9-12-r182

**Published:** 2008-12-19

**Authors:** Alyson Ashe, Daniel K Morgan, Nadia C Whitelaw, Timothy J Bruxner, Nicola K Vickaryous, Liza L Cox, Natalie C Butterfield, Carol Wicking, Marnie E Blewitt, Sarah J Wilkins, Gregory J Anderson, Timothy C Cox, Emma Whitelaw

**Affiliations:** 1 Epigenetics Laboratory, Queensland Institute of Medical Research, 300 Herston Road, Herston, Queensland 4006, Australia; 2 School of Medicine, University of Queensland, Brisbane, 4001, Australia; 3 Division of Craniofacial Medicine, Department of Pediatrics, University of Washington, Seattle 98195, WA, USA; 4 Institute for Molecular Bioscience, The University of Queensland, St Lucia, Queensland 4072, Australia; 5 Walter and Eliza Hall Institute, Melbourne, Victoria 3050, Australia; 6 Iron Metabolism Laboratory, Queensland Institute of Medical Research, 300 Herston Road, Herston, Queensland 4006, Australia

## Abstract

**Background:**

Some years ago we established an N-ethyl-N-nitrosourea screen for modifiers of transgene variegation in the mouse and a preliminary description of the first six mutant lines, named *MommeD1-D6*, has been published. We have reported the underlying genes in three cases: *MommeD1 *is a mutation in *SMC hinge domain containing 1 *(*Smchd1*), a novel modifier of epigenetic gene silencing; *MommeD2 *is a mutation in *DNA methyltransferase 1 *(*Dnmt1*); and *MommeD4 *is a mutation in *Smarca 5 *(*Snf2h*), a known chromatin remodeler. The identification of *Dnmt1 *and *Smarca5 *attest to the effectiveness of the screen design.

**Results:**

We have now extended the screen and have identified four new modifiers, *MommeD7*-*D10*. Here we show that all ten *MommeD*s link to unique sites in the genome, that homozygosity for the mutations is associated with severe developmental abnormalities and that heterozygosity results in phenotypic abnormalities and reduced reproductive fitness in some cases. In addition, we have now identified the underlying genes for *MommeD5 *and *MommeD10*. *MommeD5 *is a mutation in *Hdac1*, which encodes histone deacetylase 1, and *MommeD10 *is a mutation in *Baz1b *(also known as *Williams syndrome transcription factor*), which encodes a transcription factor containing a PHD-type zinc finger and a bromodomain. We show that reduction in the level of Baz1b in the mouse results in craniofacial features reminiscent of Williams syndrome.

**Conclusions:**

These results demonstrate the importance of dosage-dependent epigenetic reprogramming in the development of the embryo and the power of the screen to provide mouse models to study this process.

## Abstract 2

An extended ENU screen for modifiers of transgene variegation identified four new modifiers, MommeD7-D10.

## Background

Random mutagenesis screens for modifiers of position effect variegation were initiated in *Drosophila *in the 1960s [1,2]. The screens used a fly strain, called *w*^*v*^, that displays variegated expression of the *white *(*w*) locus resulting in red and white patches in the eye. Variegation refers to the 'salt and pepper' expression of some genes due to the stochastic establishment of an epigenetic state at their promoters. The best characterized example of variegation in mammals is the coat color of mice carrying the *A*^*vy *^allele [3,4]. In this case there is a correlation between DNA methylation at the promoter and silencing of the gene [5,6]. Alleles of this type provide us with an opportunity to study epigenetic gene silencing at a molecular level in a whole organism.

The extent of the variegation at the *w*^*v *^locus, that is, the ratio of red to white patches in the eye, was known to be sensitive to strain background, suggesting the existence of genetic modifiers. Offspring of mutagenized flies were screened for changes in the degree of variegation. These screens have been continued to saturation and the results suggest that there are between 100 and 150 such genes [7,8]. Approximately one-third of these have been identified and, as expected, most turn out to play critical roles in epigenetic gene silencing [1,9]. These include genes encoding proteins involved in heterochromatin formation, for example, HP1 and histone methyltransferases [8].

Recently, we established a similar screen in the mouse using a transgenic line that expresses green fluorescent protein (GFP) in a variegated manner in erythrocytes [10]. We anticipated that the screen would produce mutant lines that would help clarify the role of epigenetic gene silencing in mammals. Offspring of N-ethyl-N-nitrosourea (ENU) treated males were screened for changes in the percentage of erythrocytes expressing GFP (measured by flow cytometry). In those individuals in which the percentage of expressing cells was higher or lower than the wild-type mean by more than two standard deviations, heritability was tested. A preliminary description of the first six heritable mutations, which we refer to as *Modifiers of murine metastable epialleles *or *Mommes*, following the screening of 608 G1 offspring, has been published [10]. We reported that all six were dosage-effect genes and five of the six were homozygous lethal, with loss of homozygotes apparent at weaning, but no knowledge of when death occurred. At the time of publication in 2005, none of the underlying genes had been identified. Since then we have identified the underlying mutation in three cases, *MommeD1*, *MommeD2 *and *MommeD4*. *MommeD1 *is a mutation in *SMC hinge domain containing 1 *(*SmcHD1*), encoding a previously uncharacterized protein containing a domain normally found in SMC proteins and we have gone on to show that this protein has a critical role in X inactivation [11]. *MommeD2 *is a mutation in *DNA methyltransferase 1*, *Dnmt1*, encoding a DNA methyltransferase, and *MommeD4 *is a mutation in *Smarca5*, encoding Snf2h, a chromatin-remodeling enzyme [12]. The finding of *Dnmt1 *and *Smarca5*, both known to be involved in epigenetic reprogramming, validates the screen. Here we report an update of the screen, adding four new *MommeD*s, identifying the underlying point mutation in two more cases, and further characterizing the phenotypes associated with hetero- and homozygosity.

## Results and discussion

### Integration site of the GFP transgene

We have previously reported that the GFP transgene used in this screen has integrated as an approximately 11 copy array on chromosome 1 [10]. We were keen to further characterize the integration site. PCR using primers specific to the 3' end of the transgene construct in combination with degenerate random tagging primers (genome walking) revealed that the transgene had integrated into chromosome 1 at 47,747,486 bp (UCSC web browser, July 2007 assembly). This site of integration is neither centromeric nor telomeric, and so presumably the silencing is related to the multicopy nature of the transgene array [13,14]. The integration site does not disrupt any annotated genes, and is approximately 1 Mb from the closest annotated transcript.

### The identification of *MommeD7-D10*

**Figure 1:**
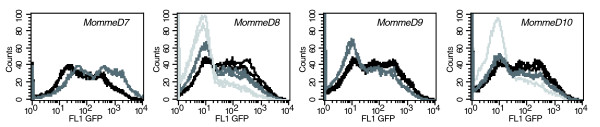
**GFP expression profiles in *MommeD7-D10***. Erythrocytes from 3-week-old mice were analyzed by flow cytometry. In each case, the expression profiles from one litter of a heterozygous intercross are displayed. The phenotypically wild-type mice are shown in black, heterozygotes in dark grey, and homozygotes (*MommeD8 *and *D10 *only) in light grey. The x-axis represents the erythrocyte fluorescence on a logarithmic scale, and the y-axis is the number of cells detected at each fluorescence level. For quantitative and statistical significance, see Table 1.

We have now screened an additional 400 G1 offspring and recovered four more *Momme*s, *MommeD7-D10 *(Figure 1, Table 1). The fluorescence activated cell sorting (FACS)-based screening is carried out on a drop of blood taken at weaning, using a gate set to exclude 99.9% of autofluorescing cells. Under these conditions, wild-type mice homozygous for the transgene express GFP in 55% of erythrocytes. *MommeD7 *is a suppressor of variegation, that is, the percentage of cells expressing the transgene was significantly higher than it was in wild-type individuals (Table 1). *MommeD8*, *D9 *and *D10 *are enhancers of variegation, that is, the percentages of expressing cells were significantly lower than they were in wild-type individuals (Table 1). The mean fluorescence level in expressing cells also changed. We have reported previously that as the percentage of expressing cells increases, the mean fluorescence of the expressing cells increases [10]. We presume that since the mice are homozygous for the GFP transgene, this is mainly due to an increase in the proportion of expressing cells with two active GFP alleles. However, in the case of *MommeD7 *the level was more than double that seen in the wild-type littermates. We hypothesized that this was likely to be the consequence of an increase in the percentage of reticulocytes in the peripheral blood of this mutant, as mature red cells, with no ability to produce new proteins, would have, on average, less GFP than reticulocytes (see below). In the cases of *MommeD8*, *D9 *and *D10 *the mean fluorescence levels were slightly lower than that seen in the wild-type littermates, consistent with a presumed reduction in the proportion of cells with two active GFP alleles.

**Table 1:** Quantitative analysis of GFP expression following heterozygous intercrosses

	Percentage expressing cells			Mean fluorescence		
		
Mutant	WT (n)	Heterozygote (n)	Homozygote (n)	WT (n)	Heterozygote (n)	Homozygote (n)
*MommeD7*	55 ± 7 (7)	80 ± 3* (13)	NA	286 ± 43 (7)	700 ± 52* (13)	NA
*MommeD8*	54 ± 4 (40)	41 ± 4* (55)	15 ± 4* (15)	287 ± 25 (40)	244 ± 28* (55)	161 ± 27* (15)
*MommeD9*	59 ± 4 (8)	39 ± 6* (13)	NA	326 ± 37 (8)	228 ± 27* (13)	NA
*MommeD10*	55 ± 4 (46)	44 ± 3* (87)	22 ± 2* (6)	295 ± 33 (46)	269 ± 26* (87)	243 ± 19* (6)

The percentage of expressing cells was determined by using a GFP^+ ^gate, which was set to exclude 99.9% of wild-type (WT) cells. Data were collected from at least four litters in each case. The data for the *MommeD9 *colony were collected using a different laser. Each mutant line has a significantly different expression profile to that of wild-type littermates, reproducible over many generations. *p ≤ 0.0001.

For each *MommeD*, the heritability of the mutation has been tested and confirmed over at least 5 generations by using at least 30 litters. During the breeding of each mutant line, the expression profiles remained constant, consistent with the idea that we were following a single mutation in each case. The frequency with which we found these mutations, 1 in 100 G1 offspring, was similar to our previous results [10].

### Homozygous lethality

**Figure 2:**
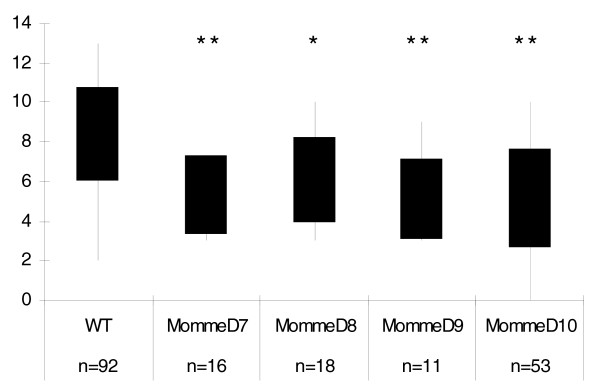
**Litter size at weaning following heterozygous intercrosses**. Litter size at weaning in all of *MommeD7-D10 *is significantly lower than that found in a wild-type (WT) cross. n represents the number of litters. *p < 0.05; **p < 0.001.

Following heterozygous intercrosses, the proportion of expression types observed in the offspring at weaning was consistent with a semidominant homozygous lethal mutation in the cases of *MommeD7 *and *MommeD9*, since only two GFP expression profiles were observed (Figure 1, Tables 1 and 2) and there was a significant litter size reduction in both cases (Figure 2). In the cases of *MommeD8 *and *MommeD10*, three expression profiles were observed, suggesting viability of some homozygotes (Figure 1, Tables 1 and 2) and in the case of *MommeD10 *this was later confirmed by genotyping for the point mutation. In both cases, fewer individuals with the severe phenotypes were observed than predicted by Mendelian inheritance, suggesting reduced viability of the homozygotes (Table 2). This conclusion is supported by significant litter size reductions in both cases (Figure 2). There is also a suggestion of some heterozygous death in the case of *MommeD8 *and, to a lesser extent, *MommeD10 *but this is not statistically significant.

**Table 2:** Genotype ratios of offspring at weaning following heterozygous intercrosses

	Observed	Expected if semidominant, homozygous viable	Expected if semidominant, homozygous lethal
*MommeD7*			
Heterozygote*	65		60
Wild type	25		30
			
*MommeD8*			
Homozygote*	14	27.5	
Heterozygote*	56	55	
Wild type	40	27.5	
			
*MommeD9*			
Heterozygote*	31		37.3
Wild type	25		18.7
			
*MommeD10*			
Homozygote* ^†^	14	77.25	
Heterozygote* ^†^	187	154.5	
Wild type	108	77.25	

Offspring were classified as wild type, heterozygous or homozygous based on their GFP expression profiles. *p*-values: *MommeD7*, not significant; *MommeD8*, *p *< 0.005; *MommeD9*, not significant; *MommeD10*, *p *< 0.0001. *Progeny testing of mice classified based on GFP expression was carried out in a number of cases: 5 for *MommeD7*^-/+^, 11 for *MommeD8*^-/-^, 16 for *MommeD8*^-/+^, 8 for *MommeD9*^-/+^, 6 for *MommeD10*^-/- ^and 11 for *MommeD10*^-/+^. In all cases the genotype was as expected. ^†^Presumed genotype was confirmed by genotyping for 8 *MommeD10*^-/- ^and 79 *MommeD10*^-/+ ^mice.

### Homozygous lethality occurs at different stages of development

Litter size reductions following heterozygous intercrosses have already been reported for *MommeD1-D6 *at weaning [10], but the timing of the losses has only been reported for *MommeD1*, *D2* and *D4*. *MommeD1 *is homozygous lethal in females only, with death occurring around mid-gestation [10,11]. *MommeD2 *and *MommeD4 *are homozygous lethal at 8.5 days post-coitus (dpc) and 17.5 dpc, respectively [10,12]. Here we describe the timing of the losses for *MommeD3*, *D5*, *D6*, *D7*, *D8*, *D9 *and *D10*.

Following intercrosses between *MommeD3*^-/+ ^(genotypes determined by GFP fluorescence and progeny testing), dissections at 14.5 dpc suggested that death of homozygotes had already occurred (Table 3). This was confirmed following a FVB/C57 F_1 _*MommeD3*^-/+ ^intercross, where embryos could be genotyped using microsatellite markers across the linked interval (Table 3). These data suggest *MommeD3*^-/- ^mice were dying at or before 14.5 dpc. Similar results were obtained for *MommeD5 *at 14.5 dpc (Table 3). Once the *MommeD5 *point mutation had been found (see below), these crosses were repeated and dissections were performed at 10.5 dpc. Again, a significantly higher than expected proportion of developmentally delayed embryos were detected (Table 3). These embryos were genotyped and found to be *MommeD5*^-/- ^in all cases, indicating developmental arrest at around 8-9 dpc. Results obtained for *MommeD6 *(genotypes determined by GFP fluorescence and progeny testing) were similar (Table 3), suggesting *MommeD6*^-/- ^embryos arrest around 8-9 dpc.

**Table 3:** Embryo dissections to determine time of death of homozygotes

	10.5 dpc		14.5 dpc		17.5/18.5 dpc	
			
Name	Normal	Abnormal or resorbed	Normal	Abnormal or resorbed	Normal	Abnormal or resorbed
*MommeD3*			48 (60%)	32 (40%)*		
*MommeD3 *(*F1*)			80 (74%)	28 (26%)* ^†^		
*MommeD5*	35 (74%)	12 (26%)* ^‡^	42 (72%)	16 (28%)*		
*MommeD6*	38 (63%)	22 (37%)*	25 (62.5%)	15 (37.5%)*		
*MommeD7*			44 (85%)	8 (15%)^§^	63 (74%)	22 (26%)
*MommeD8*			101 (94%)	7 (6%)		
*MommeD9*	16 (64%)	9 (36%)*	19 (59%)	13 (41%)*		
*MommeD10*			54 (93%)	4 (7%)	86 (88%)^¶^	12 (12%)^¥^
Control cross	34 (94%)	2 (6%)	266 (93%)	19 (7%)		

^†^The abnormal embryos from which DNA could be extracted (17) were genotyped across the linked interval and all shown to be *MommeD3*^-/-^. ^‡^These abnormal (very small) embryos were genotyped and shown to be *MommeD5*^-/-^. ^¶^These normal embryos were genotyped and found to have the expected ratio of *MommeD10*^+/+^, *MommeD10*^-/+ ^and *MommeD10*^-/- ^embryos. ^¥^Four of the abnormal embryos were able to be genotyped: two were *MommeD10*^-/+ ^and two were *MommeD10*^-/-^. *p < 0.0001; ^§^*p *< 0.05.

**Figure 3:**
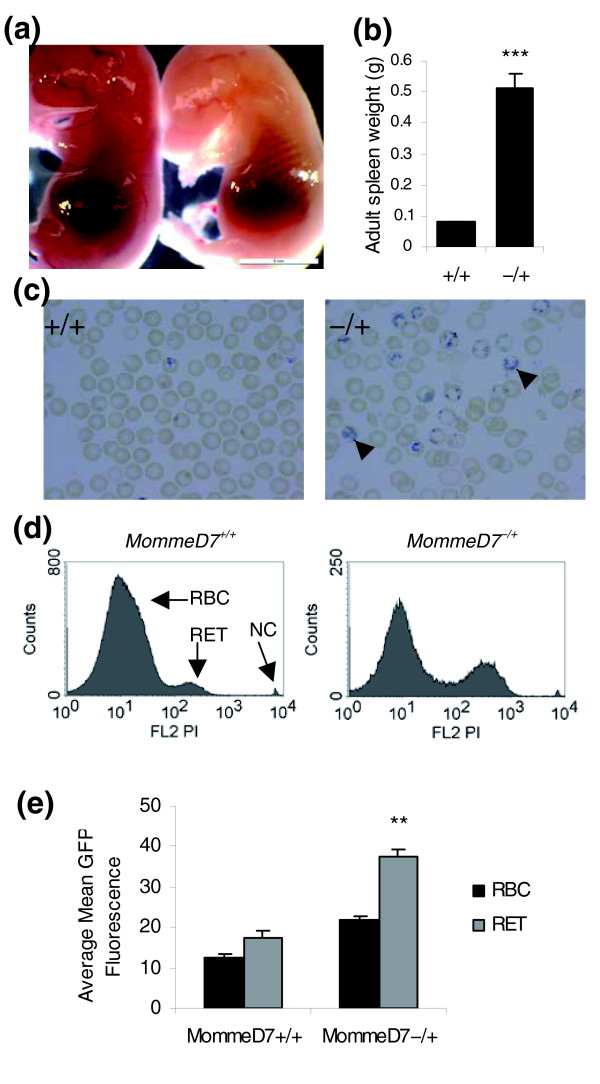
**Hematopoietic abnormalities in *MommeD7 *mice**. **(a) **Examples of phenotypically normal and abnormal (pale) embryos at 17.5 dpc. Abnormal embryos are assumed to be homozygous for *MommeD7*. Scale bar = 5 mm. **(b) **Spleen weights from *MommeD7*^+/+ ^and *MommeD7*^-/+ ^adult mice. Error bars represent SEM. ***p < 0.0001. **(c) **Blood smears from *MommeD7*^+/+ ^(left) and *MommeD7*^-/+ ^(right) mice stained for reticulocytes (shown with arrowheads). **(d) **Representative histograms showing propidium iodide fluorescence in *MommeD7*^+/+ ^(left) and *MommeD7*^-/+ ^(right) mice. In each case 10,000 reticulocytes were counted. Red blood cells (RBC), reticulocytes (RET) and nucleated cells (NC) are shown. **(e) **Histogram showing relative levels of GFP fluorescence in red blood cells and reticulocytes. Averages were calculated from three wild-type and three heterozygous mice. **p < 0.005; error bars represent SEM.

Following *MommeD7*^-/+ ^intercrosses (genotypes determined by GFP fluorescence and progeny testing), a small but significant increase in abnormal embryos was detected at 14.5 dpc (Table 3). This increase is not enough to account for all expected *MommeD7*^-/- ^mice. At 17.5 dpc, approximately one-quarter of the embryos were pale (Table 3, Figure 3a), suggesting a red cell defect in the homozygotes. Homozygous *MommeD7 *mutants were never seen at weaning (Table 2), and preliminary observations suggest that they die in the first few days after birth. Further analysis of adult heterozygous individuals revealed severe splenomegaly (Figure 3b) and a marked increase in reticulocytes in peripheral blood (Figure 3c).

We hypothesized that this increase in reticulocytes was responsible for the larger than expected increase in the average fluorescence level of the GFP transgene in expressing cells observed in this line (Table 1). We performed FACS analysis on whole blood after staining for reticulocytes with propidium iodide. As expected, *MommeD7*^-/+ ^mice had a threefold increase in the percentage of reticulocytes compared to *MommeD7*^+/+ ^mice (Figure 3d), and the percentage of GFP fluorescence in both *MommeD7*^-/+ ^and *MommeD7*^+/+ ^was higher in reticulocytes than mature red cells (Figure 3e). Although this is only significant for *MommeD7*^-/+ ^(*p *< 0.005), the trend is there for *MommeD7*^+/+ ^mice (*p *= 0.07). This is consistent with the idea that a change in the erythroid cell populations contributes to the dramatic increase in the average fluorescence level of the GFP transgene in *MommeD7*^-/+ ^mice.

Some *MommeD8*^-/- ^mice (classified by their GFP expression profile and progeny testing) were viable at weaning but they were rare (Figure 1, Table 2). Following *MommeD8*^-/+ ^intercrosses, dissections at 14.5 dpc showed no increase in the number of abnormal or resorbed embryos (Table 3). Litter size at birth was not significantly different from that seen in wild-type litters (data not shown), suggesting that the death of most *MommeD8*^-/- ^individuals occurred after birth and before weaning. The only obvious phenotypic abnormality seen in *MommeD8*^-/- ^mice that survived to weaning was reduced size. *MommeD8 *homozygotes were significantly smaller (6.60 g ± 0.25 standard error of the mean (SEM)) than their wild-type (8.54 g ± 0.33 SEM, *p *< 0.001) and heterozygous (8.65 g ± 0.29 SEM, *p *< 0.0001) littermates.

Dissections following *MommeD9*^-/+ ^(determined by GFP fluorescence and progeny testing) intercrosses revealed a pattern similar to that seen for *MommeD5 *and *MommeD6*, suggesting *MommeD9*^-/- ^embryos arrest before 9.5 dpc (Table 3). In the case of *MommeD10 *the data suggest that death of homozygotes occurred after birth (Table 3), and preliminary data collected at P7 indicated death in the first week of life (data not shown). Some *MommeD10*^-/- ^individuals survived to weaning but they were extremely rare. This was confirmed by genotyping once the point mutation was identified.

So in all ten *MommeD*s produced so far, homozygosity for the mutation is associated with embryonic or perinatal lethality (Tables 3 and 4).

**Table 4:** Summary of *MommeD *screen for epigenetic modifiers

Name	Suppressor or enhancer of variegation	Mapping (Mb)	Mutated gene, or number of genes in interval	Homozygous lethality
*MommeD1*	Suppressor	Chr 17 (2.4)	SmcHD1*	Females only E10
*MommeD2*	Suppressor	Chr 9 (1.4)	*Dnmt1*^†^	E9
*MommeD3*	Suppressor	Chr 11 (6)	123	E9-E14
*MommeD4*	Enhancer	Chr 8 (2.5)	*Smarca5*^†^	E17
*MommeD5*	Enhancer	Chr 4 (1.4)	*Hdac1*	E8-E9
*MommeD6*	Suppressor	Chr 14 (2.5)	32	E8-E9
*MommeD7*	Suppressor	Chr 7 (0.25)	10	P1-P7
*MommeD8*	Enhancer	Chr 4 (4)	54	Most P1-P21
*MommeD9*	Enhancer	Chr 7 (4)	80	E9
*MommeD10*	Enhancer	Chr 5 (4)	*Baz1b*	Most P1-P21

All mapping data are current for Ensembl build 37.1, release 49. *Reported in [11]. ^†^Reported in [12].

### Abnormal phenotypes associated with heterozygosity for *MommeD7-D10*

**Figure 4:**
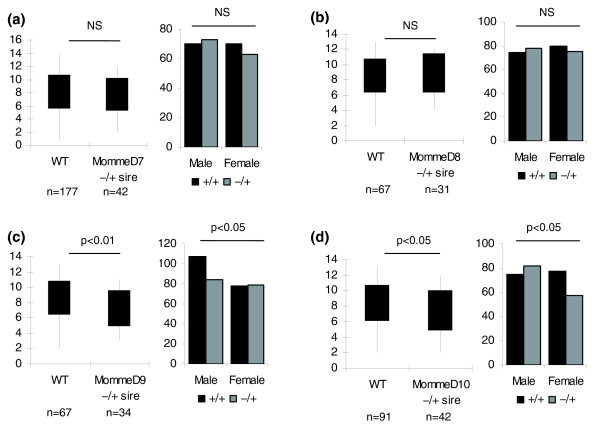
**Genotypes and sex of offspring, and litter size following paternal transmission of *MommeD7-D10***. **(a) ***MommeD7*, **(b) ***MommeD8*, **(c) ***MommeD9*, and **(d) ***MommeD10 *show the numbers of male and female offspring of wild-type (WT; black) and heterozygous (grey) genotype produced following a cross between a male heterozygous *Momme *and a wild-type female. *MommeD9 *and *MommeD10 *both show a trend towards transmission ratio distortion and a significant reduction in litter size compared to wild-type crosses. n represents the number of litters produced.

Extensive phenotyping of the heterozygous *MommeD *mutant lines has not been carried out. However, in some cases heterozygous effects were obvious, for example, the haematopoietic defect in *MommeD7*^-/+ ^mice described above. We have also noticed some litter size reduction during the breeding of these strains. The data for the breeding of *MommeD7*, *D8*, *D9 *and *D10 *are shown in Figure 4. Following crosses between heterozygous males and wild-type females in the FVB strain, we found significant litter size reductions in the cases of *MommeD9 *and *MommeD10*, but not in the cases of *MommeD7 *and *MommeD8*. A breakdown of the offspring by sex and genotype revealed that for *MommeD9 *and *MommeD10*, the litter size reduction was associated with a deviation from Mendelian patterns of inheritance (*p *< 0.05 in both cases) and a reduction in the number of mutants (Figure 4). These two cases of transmission ratio distortion have not been investigated further but they do suggest that heterozygosity for the *MommeD *mutations is associated with some level of disadvantage. There also appears to be a skewed sex ratio in the wild-type offspring of *MommeD9 *sires, suggesting the phenotype of the father can affect his wild-type offspring. While we have not characterized this in any more detail, the idea of a paternal effect is not new. We have previously published examples of paternal effects resulting from haploinsufficiency of modifiers of epigenetic gene silencing in the mouse [12].

### Mapping

We have mapped the mutations in all ten cases to relatively small regions of the genome (Table 4). The mapping of *MommeD1-D6 *has been documented [10]. Here we report the mapping of *MommeD7-10*. *MommeD7 *maps to a 0.25 Mb region on chromosome 7 between markers D7Mit220 and rs13479441 (using 134 phenotypically mutant and 135 phenotypically wild-type mice). This region contains 10 genes. *MommeD8 *maps to a 4 Mb region on chromosome 4 between markers rs6337795 and D4Mit279 (using 234 phenotypically mutant and 177 phenotypically wild-type mice). This region contains 54 genes. *MommeD9 *maps to a 3 Mb region on chromosome 7 between markers rs31712695 and rs6193818 (using 103 phenotypically mutant and 127 phenotypically wild-type mice). This region contains 80 genes. *MommeD10 *maps to a 4 Mb region between markers D5Mit165 and rs13478547 on chromosome 5. Twenty-four phenotypically homozygous and 312 phenotypically non-homozygous (heterozygous and wild-type mice combined) were used (see Materials and methods). These data show that each of the ten *MommeD *mutations maps to a unique region of the genome.

### *MommeD5 *has a mutation in *Histone deacetylase 1*

**Figure 5:**
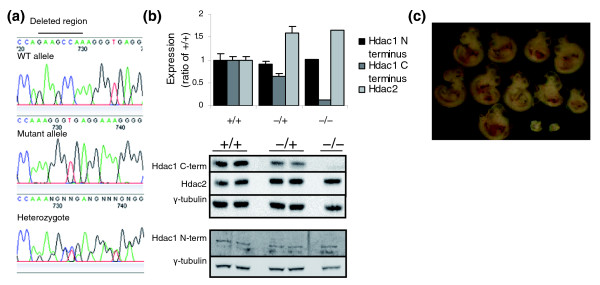
**A mutation in *Hdac1 *correlates with the *MommeD5*^-/- ^phenotype**. **(a) **A 7 bp deletion was detected in exon 13 of *Hdac1*. Representative chromatograms from the wild-type (WT) allele, the mutant allele, and one heterozygote are shown. This deletion alters the reading frame, changing the last 12 amino acids and adding 25 extra amino acids. **(b) **Whole-cell lysates from six individual 10.5 dpc *MommeD5*^+/+ ^and *MommeD5*^-/+ ^embryos, and six pooled *MommeD5*^-/- ^embryos were probed with antibodies to the Hdac1 carboxyl terminus (top panel), Hdac2 (top panel) and Hdac1 amino terminus (bottom panel). Anti-γ-tubulin was used as a loading control in each case. Quantification of the Hdac1 carboxyl terminus relative to γ-tubulin shows negligible binding in *MommeD5*^-/- ^mice. Quantification of Hdac2 levels relative to γ-tubulin shows increased Hdac2 in *MommeD5*^-/+ ^and *MommeD5*^-/- ^embryos. Quantification of Hdac1 amino terminus relative to γ-tubulin shows equal levels of Hdac1 in all mice. A peptide blocking experiment was performed to confirm band identity. A representative western blot is shown. Error bars represent SEM. **(c) **A representative litter from a *MommeD5*^-/+ ^intercross at 10.5 dpc. *MommeD5*^-/- ^embryos (bottom right) are always dramatically smaller than *MommeD5*^+/+ ^and *MommeD5*^-/+ ^littermates.

*MommeD5 *was localized to a 1.42 Mb region on chromosome 4 flanked by the markers rs27486641 and rs27541967 [10] (Table 4). This interval contains 46 genes and *Hdac1 *was chosen as the best candidate because of its known role in chromatin modification. Sequencing of all exons, including exon/intron boundaries, from two heterozygous and two wild-type individuals revealed a 7 bp deletion (GAAGCCA) in exon 13 in the mutants (Figure 5a). This mutation was subsequently verified in over 100 mice classified as mutants by GFP expression profiling. The chance of a second point mutation occurring in a functional region in a linked interval of this size is extremely small. Using the estimated mutation rate following ENU mutagenesis of 1 in 1.82 Mb [15,16], the probability of such an event can be calculated [15,17]. This website takes into account the amount of coding sequence in a given linked interval. In this case, the probability of a second point mutation occurring in the coding region is *p *< 0.0005.

The mutation produces a frameshift, resulting in changes to the last 12 amino acids, and an additional 25 amino acids. Protein modeling predictions based on human HDAC8, for which the crystal structure has been solved [18,19], suggest that the carboxyl terminus of Hdac1 is relatively unstructured and so the mutation is unlikely to affect the stability of the protein (J Matthews, personal communication). An antibody that recognizes the carboxyl terminus of Hdac1 showed a 50% reduction of binding in 10.5 dpc *MommeD5*^-/+ ^embryos, and negligible binding in *MommeD5*^-/- ^embryos (Figure 5b), confirming that this region of the protein has been altered in the *MommeD5 *line. An antibody that recognizes the amino terminus of Hdac1 showed that the levels of the protein are not altered between mutant and wild-type mice (Figure 5b). Lysine 476 at the carboxyl terminus has been shown to be sumoylated and important for enzymatic function of the wild-type protein [20] and the absence of this amino acid in the *MommeD5 *mutant protein is likely to impair function. A knockout of *Hdac1 *has been reported and the homozygous embryos died around 9.5 dpc [21], similar to the time of death observed in *MommeD5*^-/- ^embryos (Figure 5c). Together, these results argue that the mutation in *Hdac1 *is causative of the *MommeD5 *phenotype. Consistent with this, the level of Hdac2 increased in both *MommeD5*^-/+ ^and *MommeD5*^-/- ^embryos, as predicted from the reports of compensatory upregulation of Hdac2 in embryonic stem cells null for Hdac1 [21]. Indeed, this upregulation may explain why *MommeD5 *was identified as an enhancer, rather than a suppressor, of variegation. Loss of histone deacetylase function is generally associated with transcriptional activation, but exceptions to this have been reported and the upregulation of Hdac2 could explain these results [22].

### *MommeD10 *has a mutation in *Baz1b*

**Figure 6:**
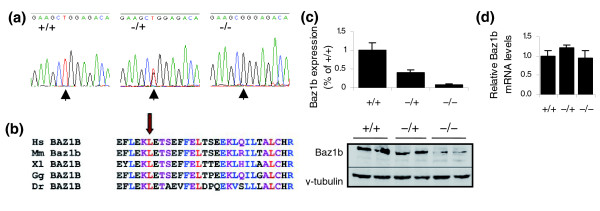
**A point mutation in *Baz1b *correlates with the *MommeD10*^-/- ^phenotype**. **(a) **A single base-pair mutation was detected in exon 7 of *Baz1b*. Representative chromatograms from one wild type, one heterozygote and one homozygote are shown. This results in a non-conservative amino acid change, L733R. **(b) **The *MommeD10 *point mutation (arrow) modifies an amino acid highly conserved across species (Hs, *Homo sapiens*; Mm, *Mus musculus*; Xl, *Xenopus laevis*; Gg, *Gallus gallus*; Dr, *Danio rerio*). **(c) **Whole-cell lysates from four 14.5 dpc heads of each genotype were probed with anti-Baz1b. Anti-γ-tubulin was used as a loading control. Quantification of Baz1b relative to γ-tubulin shows negligible levels of Baz1b in *MommeD10*^-/- ^mice. A representative western blot is shown. **(d) **Expression analysis by quantitative real-time RT-PCR was performed on mRNA prepared from 14.5 dpc heads. Levels of *Baz1b *mRNA were not affected by the mutation. Analysis was performed on three individuals of each genotype and normalized to two house-keeping genes. Error bars in all graphs represent SEM.

*MommeD10 *was localized to a 4 Mb region on chromosome 5 flanked by the markers D5Mit165 and rs32250347 (Table 4). Interestingly, this interval encompasses the region syntenic with the Williams Beuren syndrome (WBS) critical region in humans. WBS, also known as Williams syndrome, is an autosomal dominant disorder affecting approximately 1 in 10,000 individuals. The classic presentation of WBS includes a characteristic craniofacial dysmorphology (elfin face), supravalvular aortic stenosis, multiple peripheral pulmonary arterial stenoses, statural deficiency, infantile hypocalcaemia and a distinct cognitive profile with mild mental retardation. The linked interval for *MommeD10 *contains 52 genes and *Baz1b *was chosen as the best candidate because it contains a bromodomain (a domain commonly associated with chromatin remodelers) and has recently been shown to form at least two chromatin remodeling complexes and associate with replication foci and promoters [23-25]. Sequencing of all exons, including exon/intron boundaries, from two homozygous, one heterozygous and one wild-type individual revealed one point mutation (T → G transversion) in exon 7 in the mutants (Figure 6a). This mutation was subsequently verified in over 100 mice classified as mutants by GFP expression profile. The mutation results in a non-conservative amino acid change, L733R, in a highly conserved region of the protein (Figure 6b). Western blot analysis showed reduced levels of Baz1b protein in both embryonic and adult *MommeD10*^-/- ^tissue, with *MommeD10*^-/+ ^tissue showing intermediate levels (Figure 6c and data not shown), suggesting that the mutant protein is much less stable than its wild-type counterpart. Quantitative real-time PCR performed on cDNA from 14.5 dpc embryos showed all three genotypes have similar levels of mRNA (Figure 6d).

### Effects of *MommeD5 *and *MommeD10 *on DNA methylation at the transgene locus

**Figure 7:**
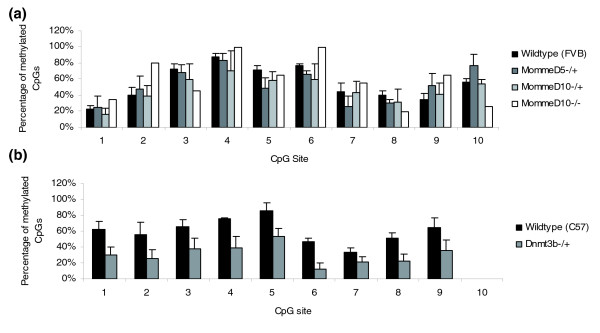
**Bisulfite analysis of the HS-40 enhancer element**. **(a) **DNA was extracted from the spleen of 4-week-old wild-type (FVB/NJ; three mice), *MommeD5*^-/+ ^(three mice), *MommeD10*^-/+ ^(three mice) mice and from the tail of *MommeD10*^-/- ^mice (two mice). After bisulfite sequencing, the percentage of CpG methylation at each CpG site in the cloned region was analyzed, and averaged across individuals of the same genotype. None are significantly different from the wild-type mouse. Error bars represent SEM. **(b) **DNA was extracted from the tail of 4-week-old wild-type (C57BL/6; four mice) and *Dnmt3b*^+/- ^(four mice) mice. After bisulfite sequencing, the percentage of CpG methylation at each CpG site in the cloned region was analyzed, and averaged across individuals of the same genotype. The *Dnmt3b*^+/- ^mice have significantly (*p *< 0.001) less methylation than the wild-type mice. Error bars represent SEM.

Transgene silencing can be associated with changes in both DNA methylation [26,27] and chromatin accessibility [28]. This particular transgene promoter consists of a GC-rich segment of the human alpha-globin promoter, which we were unable to analyze by bisulfite sequencing because the cloned bisulfite converted fragment was refractory to growth in bacteria. The transgene also contains the HS-40 enhancer, which is known to regulate the locus in humans [29]. We analyzed the methylation state at this region by bisulfite sequencing. As predicted from the variegated nature of the transgene expression, the methylation pattern differed from clone to clone in all cases (data not shown). The percentage of methylated CpGs in the HS-40 element was approximately 55% (averaged across all clones) in spleen from 4-week-old wild-type FVB/NJ mice (Figure 7a). Samples from *MommeD5*^-/+^, *MommeD10*^-/+^, and *MommeD10*^-/- ^mice showed similar levels of CpG methylation (52%, 47%, 59% respectively; Figure 7a). Mice heterozygous for a null allele of *Dnmt3b*, which showed an increase in expression of the GFP transgene from 37 ± 3% in the wild-type mice to 55.5 ± 2.5% in the *Dnmt3b*^+/- ^mice (in both cases mice were hemizygous for the transgene; see Materials and methods), showed a decrease in CpG methylation at the HS-40 element (31%; Figure 7b) compared to that seen in the wild-type C57BL/6J mouse strain (60%; Figure 7b). These data suggest that *MommeD5 *and *MommeD10 *mutants alter the expression of the transgene by changing the chromatin state rather than by altering DNA methylation levels. This is consistent with the fact that both genes encode proteins involved in histone modification and chromatin remodeling [21,23-25,30-33]. Modifiers identified in this screen include DNA methyltransferases, chromatin remodelers and genes involved in histone modification, all of which have wide-ranging effects in the genome, making it difficult to unravel direct and indirect effects at any particular locus.

### Craniofacial analysis of *MommeD10 *mice

**Figure 8:**
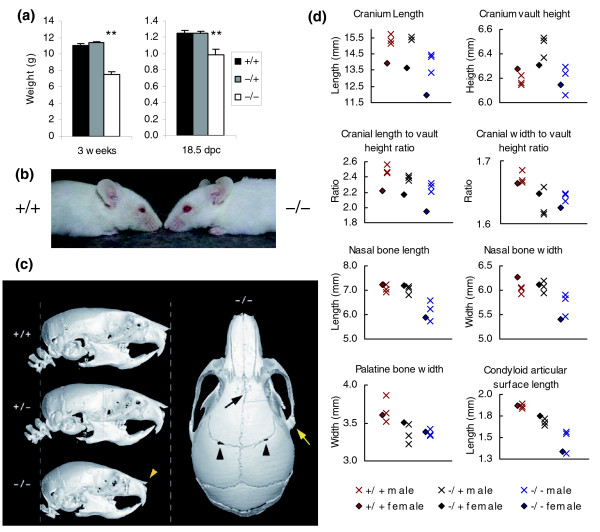
***MommeD10*^-/- ^mice are smaller than their littermates and display craniofacial abnormalities**. **(a) **Body weight was measured for 46 *MommeD10*^+/+^, 102 *MommeD10*^-/+ ^and 10 *MommeD10*^-/- ^weaners (3 weeks), and 11 *MommeD10*^+/+^, 22 *MommeD10*^-/+ ^and 5 *MommeD10*^-/- ^embryos (18.5 dpc). Histograms show mean and SEM. **(b) **Craniofacial abnormalities in adult *MommeD10*^-/- ^mice. *MommeD10*^-/- ^mice display shorter snouts than age and sex-matched wild-type littermates. **(c) **Three-dimensional reconstruction of skull microCT data from 4-week-old male mice reveals distinct anomalies in homozygous Baz1B mice. Left side: lateral views show the overall size and shape of heterozygous skulls is similar to that of wild-type skulls, whereas skulls of homozygotes were around 20% shorter. Homozygous skulls showed variable anomalies, but consistently had a bulbous appearance, and a short, flattened, or upwardly angulated nasal bone (yellow arrowhead). Slight angulation of the nasal bones was also noted in one heterozygote. Right side: dorsal view of the homozygote skull shown on the left side showing the abnormal shape and more rostral connection of the zygomatic process with the squamosal bone (yellow arrow), skewing of the midline frontal bone suture (black arrow) and minor bilateral anomalies of the frontal:parietal suture (black arrowheads). **(d) **Twenty cranial landmarks and nine mandibular landmarks (based on those of Richtsmeier [49]) were located on each of nine skulls and inter-landmark measurements recorded. The mean value of each measurement, including analysis of cranium height:width and cranium length:height ratios, was compared between homozygous, heterozygous and wild-type animals.

Surviving *MommeD10 *homozygotes were significantly smaller than littermates at weaning (Student's *t*-test, *p *< 0.0001; Figure 8a). A similar size differential was evident *in utero *at 18.5dpc (Student's *t*-test, *p *< 0.01), indicating that this is not simply due to poor post-natal feeding (Figure 8a). *MommeD10 *homozygotes also appeared to have widened, bulbous foreheads and shortened snouts (Figure 8b). To examine the craniofacial phenotype more accurately, three heads from 4-week-old male mice of each genotype (*MommeD10*^-/-^, *MommeD10*^-/+ ^and *MommeD10*^+/+^) were subjected to micro-computed tomography. Heads from one 4-week-old female of each genotype were also examined at this level. They followed the same trend as males. Visual inspection of the three-dimensional reconstructions confirmed the original observation that homozygote's skulls were more bulbous and showed a flattening of the nasal bone and upward curvature of the nasal tip (Figure 8c).

Twenty cranial landmarks and nine mandibular landmarks were located on each skull using approximately 70 micron resolution datasets and inter-landmark measurements were compared (Figure 8d and Additional data file 1). Statistical analyses were carried out using the data collected from males only. Homozygote skulls were significantly different to wild type (Student's *t*-test, length:height ratio, *p *< 0.01; width:height ratio, *p *< 0.01; length:width ratio, *p *< 0.05), confirming the bulbous appearance of the skulls on the reconstructed images. Much of this difference could be attributed to reduction of the parietal and nasal bones (both > 12.5% shorter in homozygotes compared to an overall mean length and width reduction of approximately 9%). The reduced parietal bone length and the reduction and upward angulation of the nasal bones in these mice (Figure 8c, d) are reminiscent of the decrease in the volume of the parieto-occipital region and the appearance of the nose in WBS patients [34,35]. Heterozygotes also had a decreased cranium width:height ratio (Student's *t*-test, *p *< 0.05) and decreased length:height ratio (Student's *t*-test, *p *< 0.05) compared to wild-type skulls. Of note, heterozygotes showed a reduction in palatine bone width of similar magnitude to that seen in homozygotes, suggesting a greater sensitivity of some parts of the skull to decreased Baz1b protein levels. Measurements of the lower jaw revealed relative mandibular hypoplasia in homozygotes that was most pronounced in the posterior region (approximately 20% reduction), encompassing the condyle, angle and coronoid processes (Figure 8d and Additional data file 1). The posterior aspects of the mandibles of heterozygotes were also reduced in size when compared to wild-type mandibles, albeit to a lesser degree than in the homozygotes.

### Expression of *Baz1b *during mouse embryogenesis

**Figure 9:**
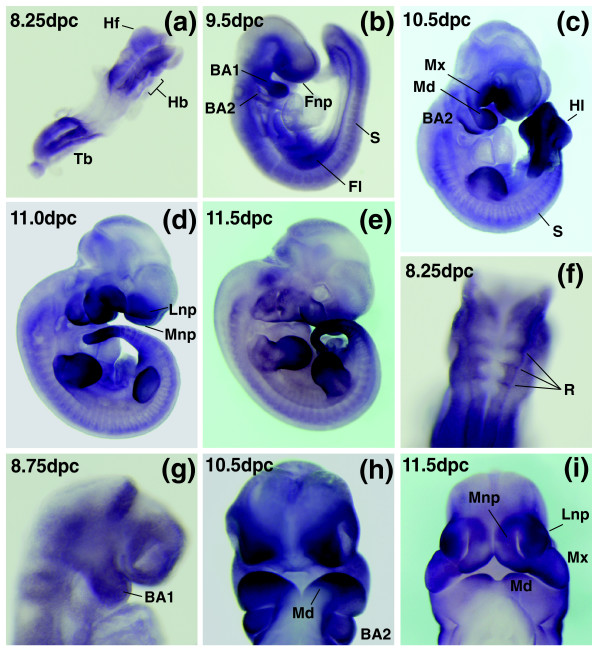
**Whole mount *in situ *hybridization analysis in mouse embryos shows expression in the developing facial prominences**. Embryos at a range of mid-gestational stages were hybridized with an antisense ribroprobe to the 3' untranslated region of *Baz1b*. **(a-e) **Views of whole embryos from 8.25-11.5 dpc reveal strong expression in the mesenchyme of the maxillary and mandibular prominences, branchial arch 2 and the nasal processes. Other sites of *Baz1b *expression include the presumptive hindbrain, headfolds and tailbud at 8.5 dpc, and the limb mesenchyme and somites at later stages. **(f) **Higher magnification dorsal view of presumptive hindbrain staining at 8.25 dpc. Close up images of facial prominence staining are shown at **(g) **8.75 dpc, **(h) **10.5 dpc, and **(i) **11.5 dpc. Images shown in (h, i) are ventral views following dissection of the head from the body of the embryo. BA1, branchial arch 1; BA2, branchial arch 2; Fl, forelimb; Fnp, frontonasal process; Hb, hindbrain; Hf, headfold; Hl, hindlimb; Lnp, lateral nasal prominence; Md, mandibular prominence; Mnp, medial nasal prominence; Mx, maxillary prominence; R, rhombomere; S, somite; Tb, tailbud.

It has previously been shown that *Baz1b *is expressed in the mouse embryo from around 9.5 dpc and whole mount *in situ *at 11.5 dpc showed expression in limb buds, tail and brain [24]. We have gone on to characterize the expression of *Baz1b *in more detail, and show that at 8.25 dpc *Baz1b *is expressed in the headfolds, the caudal tail bud region and the presumptive hindbrain in a pattern reminiscent of rhombomere staining (Figure 9a, f). From 9.5 dpc expression is evident in the somites and in the forelimb bud as it emerges from the lateral plate mesoderm (Figure 9b). Diffuse mesenchymal expression in both the forelimb and hindlimb continues until 12.5 dpc when it is restricted to the interdigital mesenchyme (data not shown).

In the facial primordia, *Baz1b *is expressed in branchial arch 1 as it first emerges (Figure 9g), and continues later in development in both the maxillary and mandibular components of branchial arch 1 and branchial arch 2 (Figure 9c-e, h, i). Expression in the branchial arches is primarily mesenchymal, and is enriched in the rostral distal margin of the mandible, and the caudal distal margin of branchial arch 2 (Figure 9c-e, h). *Baz1b *is also expressed in the frontonasal process (Figure 9b) and later in the mesenchyme of both the medial and lateral nasal prominences as they elevate to surround the nasal pits (Figure 9c, d, h, i). *Baz1b *is expressed strongly in all the major facial primordia from early in embryogenesis.

### A possible role for Baz1b in Williams syndrome

Overall, the skull shape in mutant animals is reminiscent of the head shape seen in WBS, including a small upturned nose with flat nasal bridge, micrognathia (or mandibular hypoplasia), malocclusion, bi-temporal narrowing and prominent forehead [34]. WBS is known to be associated with a hemizygous deletion of approximately 28 genes in humans, but which of these genes are responsible for the craniofacial phenotype remains controversial. People with atypical deletions, and varying degrees of craniofacial abnormalities, implicate both proximal and distal ends of the deletion, suggesting that more than one gene is responsible [36-41]. Tassabehji and colleagues [42] reported craniofacial defects in a transgenic (c-*myc*; Tgf-α) mouse line that harbored a chromosomal translocation fortuitously disrupting the *Gtf2ird1 *gene, the human orthologue of which resides in the WBS critical interval [43]. Mice homozygous for this transgene produced little *Gtf2ird1 *mRNA and exhibited craniofacial dysmorphism, suggesting a role for *Gtf2ird1 *in the craniofacial phenotype. Mice hemizygous for the transgene were indistinguishable from wild-type animals. Disruption of *Gtf2ird1 *in this mouse was associated with a 40 kb deletion at the site of integration, the addition of 5-10 tandem copies of a c-*myc *transgene, and translocation of the entire segment to chromosome 6 [43], providing opportunity for disruption to the expression of other genes, such as *Baz1b*, in the region. A targeted knockout of *Gtf2ird1*, produced by others, failed to find craniofacial dysmorphology or dental abnormalities [44]. We checked the sequence and expression of *Gtf2ird1 *in *MommeD10 *mutants and found no abnormalities (data not shown). The chance of a second point mutation occurring in a coding region in this linked interval is extremely small (*p *= 0.0008, based on a mutation rate of 1 in 1.82 Mb) [15-17].

Our studies show that the chromatin remodeler *Baz1b *is expressed strongly in cranial neural crest-derived mesenchyme that drives facial morphogenesis and that homozygosity for a missense mutation in *Baz1b *produces an array of craniofacial features that are similar to those that characterize the typical WBS face. Significantly, some craniofacial features are also apparent in heterozygotes. These results suggest that reduction in Baz1b protein levels contributes to the elfin facies characteristic of WBS and that WBS is, at least in part, a chromatin-remodeling factor disease.

## Conclusion

Extension of the screen has produced four new *MommeD*s, *MommeD7*-*D10*, all of which behave in a semidominant fashion, as do the six previously reported [10]. We have now identified the underlying genes in five of the ten cases, two of which, *Hdac1 *and *Baz1b*, we report here. Both are already known to be involved in epigenetic processes, further validating the screen design. In the case of *Baz1b *this is the first report of a mouse carrying a disrupted allele at this locus and we have shown a role for the protein in craniofacial development.

The screen, designed primarily to identify genes involved in the epigenetic gene silencing of foreign DNA, such as transgenes, has revealed the critical role that such genes play in embryonic and fetal development. It is interesting that at least half of the *MommeD*s are important during gastrulation. Furthermore, the identification of heterozygous effects suggests that a reduction in the amount of protein product of less than 50% has effects on the health of the mouse. One of the hallmarks of epigenetic gene silencing across all multicellular organisms from plants to humans is the stochastic nature by which they operate [45] and these studies re-emphasize the importance of probabilistic events during embryogenesis. We believe that this screen will provide new tools with which to study these processes.

## Materials and methods

### Mouse strains

Wild-type inbred C57BL/6J and FVB/NJ mice were purchased from ARC Perth. Procedures were approved by the Animal Ethics Committee of the University of Sydney and the Animal Ethics Committee of the Queensland Institute of Medical Research. The ENU screen was carried out in the FVB/NJ inbred transgenic line as described previously [10]. All *MommeD *lines were maintained in this strain unless stated otherwise. Most crosses between *MommeD *individuals were performed on individuals three generations or more removed from the *MommeD *progenitor. A total of 1,000 G1 offspring were screened at 3 weeks of age, from which ten heritable dominant mutations were identified.

The *Dnmt3b *null mice were a kind gift from En Li. They have been subsequently backcrossed for more than ten generations to the C57BL/6J strain. GFP fluorescence in *Dnmt3b*^+/- ^mice was analyzed in the F_1 _offspring of crosses between *Dnmt3b*^+/- ^mice and the FVB/NJ transgenic line, and as such each mouse was hemizygous for the transgene.

### Flow cytometry

GFP fluorescence in erythrocytes was analyzed by flow cytometry at weaning. A drop of blood was collected in Osmosol buffer (Lab Aids Pty Ltd, Narrabeen, NSW, Australia) and analyzed on a FACScan (Becton Dickinson, Franklin Lakes, NJ, USA) with excitation at 488 and 550 nm. The 488 nm channel predominantly measures GFP fluorescence and the 550 nm channel measures autofluoresence. The data were analyzed using CELL QUEST software with a GFP-positive gate set to exclude 99.9% of wild-type erythrocytes. Mean fluorescence was calculated using cells within the positive gate. Histograms depict only the GFP fluorescence channel.

### Genome walking

Genome walking was performed using the APAgene™ GOLD Genome Walking Kit (Bio S&T Inc., Montreal, Quebec, Canada) following the manufacturer's instructions. Gene specific primers used were (5'-3'): WalkA CCATATTTTCACCATACACGACA; WalkB GAGACTTTCTCATCCCCAAAACT; WalkC CCCCAAAACTTGTACCCAAA.

### Linkage analysis

For *MommeD7*, *D8* and *D9*, FVB/C57 F1 heterozygous individuals were backcrossed to C57, and linkage analysis performed on their offspring. Phenotype was assigned based on GFP fluorescence profile. A panel of microsatellite markers that differ in size between FVB and C57 were used to localize the mutation to a small area of the genome. Mice wild type for the mutation should only have C57 chromosomes at the linked interval, while mice heterozygous for the mutation should carry both FVB and C57 chromosomes.

Linkage analysis in *MommeD10 *was carried out using an FVB/C57 F1 *MommeD10*^-/+ ^intercross to produce 337 F2 individuals. *MommeD10*^-/- ^mice were distinguished from their littermates by their dramatically reduced size at weaning and their reduced GFP expression profile. Recombination events allowed the linked region to be localized to a small genomic interval. *MommeD10*^-/- ^mice should only carry FVB chromosomes at the *MommeD10 *linked region, while the remaining mice should be either FVB/C57 or C57/C57.

### Reticulocyte counts

Blood smears were made from blood taken from the tail tip of *MommeD7*^-/+ ^and *MommeD7*^+/+ ^mice, and stained with New methylene blue. Full blood analyses were done on the automated haematology analyzer Sysmex Xe-2100 (Sysmex Corporation, Woodlands Spectrum, Singapore).

### Reticulocyte analysis by FACS

Nucleated cells and reticulocytes were separated from mature erythrocytes based on propidium iodide fluorescence levels. An RNase control was performed and the presumptive reticulocyte cell population could no longer be detected. Mean GFP fluorescence was determined for reticulocyte and mature erythrocyte cell populations. This was done essentially as described in [46]. Three adult *MommeD7*^-/+ ^and three wild-type littermate controls were used. Approximately 25 μl of whole blood was collected from the tail in cold Osmosol buffer (Lab Aids Pty Ltd). Cells were fixed for 1 h at 4°C in 1% paraformaldehyde in Osmosol and then washed once in cold Osmosol. Cells were then permeabilized by adding -20°C ethanol to a cell pellet whilst vortexing, and incubated with rotation for 2.5 h at 4°C. A 40 μg/ml solution of propidium iodide was added to pelleted cells and the cells were incubated at 37°C for 30 minutes. Analysis was performed on a FACScan (Becton Dickinson). The data were analyzed using CELL QUEST software.

### Genotyping assay

Following identification of the *MommeD10 *point mutation, genotyping was carried out by *Aci*I digestion of a PCR product of exon 7 of *Baz1b *(primers available on request). The *Aci*I site is created by the *MommeD10 *point mutation. Following identification of the *MommeD5 *mutation, genotyping was carried out by PCR amplification across the deleted region, and gel electrophoresis using Ultra low-range agarose (Bio-Rad, Hercules, CA, USA).

### Protein analysis

We prepared whole-cell lysates of 10.5 dpc embryos (*MommeD5*) and 14.5 dpc heads and adult spleen (*MommeD10*) in 2-3× volume lysis buffer (0.05 M Tris, 7 M urea, 150 mM NaCl). Samples were incubated on ice for 20 minutes, sonicated, centrifuged, and the supernatant collected. Approximately 5 μg (*MommeD5*) or 10 μg (*MommeD10*) of protein was separated by SDS-PAGE on a 4-12% gradient gel (Invitrogen, Carlsbad, CA, USA) and was analyzed with antibodies to the Hdac1 amino terminus (sc-6299, Santa Cruz Biotechnology, Santa Cruz, CA, USA), Hdac1 carboxyl terminus (05-100, Millipore, Billerica, MA, USA), Hdac2 (05-814, Millipore), Baz1B (BL2067, Bethyl Laboratories, Montgomery, TX, USA) or γ-tubulin (T5192, Sigma-Aldrich, St Louis, MO, USA). Blots were quantified by normalizing the levels of Hdac1, Hdac2 or Baz1B in each lane to that of γ-tubulin. The normalized levels were then averaged across genotypes and the ratio to wild-type levels calculated. A peptide blocking experiment was carried out for the Hdac1 amino terminus using sc-6299P (Santa Cruz Biotechnology).

### RNA preparation and quantitative RT-PCR

Total RNA was extracted from 14.5 dpc embryo heads using TRI reagent (Sigma-Aldrich). cDNA was synthesized from total RNA using SuperScriptII reverse transcriptase (Invitrogen) and random hexamer primers. Quantitative RT-PCR was performed with the Platinum SYBR Green qPCR SuperMix-UDG with primers designed to span exon/intron boundaries (available on request). All reactions were performed in triplicate and normalized to both GAPDH and ribosomal highly basic 23-kDa protein (Rpl13A) [47]. The reaction was run on a Corbett Research Rotor-Gene (Qiagen, Doncaster, Vic, Australia).

### Bisulfite sequencing of the transgene HS-40 enhancer

Bisulfite sequencing was carried out as described in [48]. Oligonucleotides to the bisulfite converted HS-40 enhancer were as follows (5'-3'): GFPbisF1 AAAATAAAATTTTTGGATTGTTATTATTATAA; GFPbisF2 ATATTTGTAATTTTAGTATTTTGGGAGGTT; and GFPbisR AATCTCTACTCACTACAAACTCCATCTC. Cycling conditions were as follows: 94°C for 2 minutes for 1 cycle; 94°C for 30 s, 60°C for 30 s, 72°C for 45 s for 35 cycles; and 72°C for 6 minutes for 1 cycle.

### Micro-CT analysis

Three heads of 4-week-old male mice of each genotype, and one head of female mice of each genotype (*MommeD10*^-/-^, *MommeD10*^-/+ ^and *MommeD10*^+/+^) were scanned at 8.7 micron resolution using a SkyScan 1076 micro-computed tomography unit and the data set reduced to approximately 17 microns for three-dimensional reconstruction of the serial slices (CT Analyser software V.1.6.1.1; SkyScan, Kontich, Belgium). Twenty cranial landmarks and nine mandibular landmarks (based on those of Richtsmeier [49]) were located on each of nine skulls and inter-landmark measurements recorded using DataViewer software (V.1.3.0.0; SkyScan). To verify accuracy of the measurements, any landmarks showing marked differences between genotype groups were re-located on a separate day and the measurement repeated. The mean value of each measurement was compared between homozygotes, heterozygotes and wild-type animals.

### RNA probes and whole-mount *in situ *hybridization

DIG-labeled RNA probes were transcribed from linearized DNA templates and used in whole mount *in situ *hybridization analysis of wild-type FVB/NJ mouse embryos at a range of gestational stages. The probe was directed to 1.1 kb of the 3' untranslated region of *Baz1b*. A sense probe was used in earlier experiments to confirm specificity of the antisense probe. Whole mount *in situ *hybridization was performed as previously described [50]. Embryo images were captured digitally using an Olympus SZX12 microscope with DP Controller software (Olympus Corporation).

## Abbreviations

dpc: days post-coitus; ENU: N-ethyl-N-nitrosourea; FACS: fluorescence activated cell sorting; GFP: green fluorescent protein; *Momme*: *Modifier of murine metastable epialle*; SEM: standard error of the mean; WBS: Williams Beuren syndrome.

## Authors' contributions

AA carried out the genetic and molecular studies for *MommeD2 *and *MommeD10*, the FACS analysis on whole blood for *MommeD7 *and co-wrote the manuscript. DKM carried out the genetic and molecular studies for *MommeD5*. NCW carried out the genetic analysis on *MommeD7*, *MommeD8*, and *MommeD9 *and determined the transgene integration site. TJB carried out genetic studies on *MommeD6 *and initial analysis on *MommeD9*. NKV performed ENU mutagenesis and carried out genetic studies on *MommeD4 *and initial analysis on *MommeD7*. LLC carried out the craniofacial analysis. NCB and CW performed and interpreted the whole mount *in situ *hybridizations. MEB performed ENU mutagenesis and carried out genetic studies on *MommeD1 *and *MommeD3*. SJW and GJA performed the reticulocyte staining. TCC carried out the craniofacial analysis and interpretation. EW conceived the study, and project design, coordinated the project and together with other co-authors interpreted results and wrote the manuscript. All authors read and approved the final manuscript.

## Additional data files

The following additional data file is available with the online version of this paper. Additional data file 1 shows more craniofacial measurements between *MommeD10*^+/+^, *MommeD10*^-/+ ^and *MommeD10*^-/- ^mice.

## Supplementary Material

Additional data file 1 Craniofacial measurements between *MommeD10*^+/+^, *MommeD10*^-/+ ^and *MommeD10*^-/- ^mice. Click here for file
