# Supplementary material for: A genome-wide screen for modifiers of transgene variegation identifies genes with critical roles in development
Source: Genome Biol. 2008 Dec 19;9(12):R182. doi: 10.1186/gb-2008-9-12-r182 (PMC2646286; doi:10.1186/gb-2008-9-12-r182)
Supplement: Supplementary file 1 [file gb-2008-9-12-r182-S1.doc]

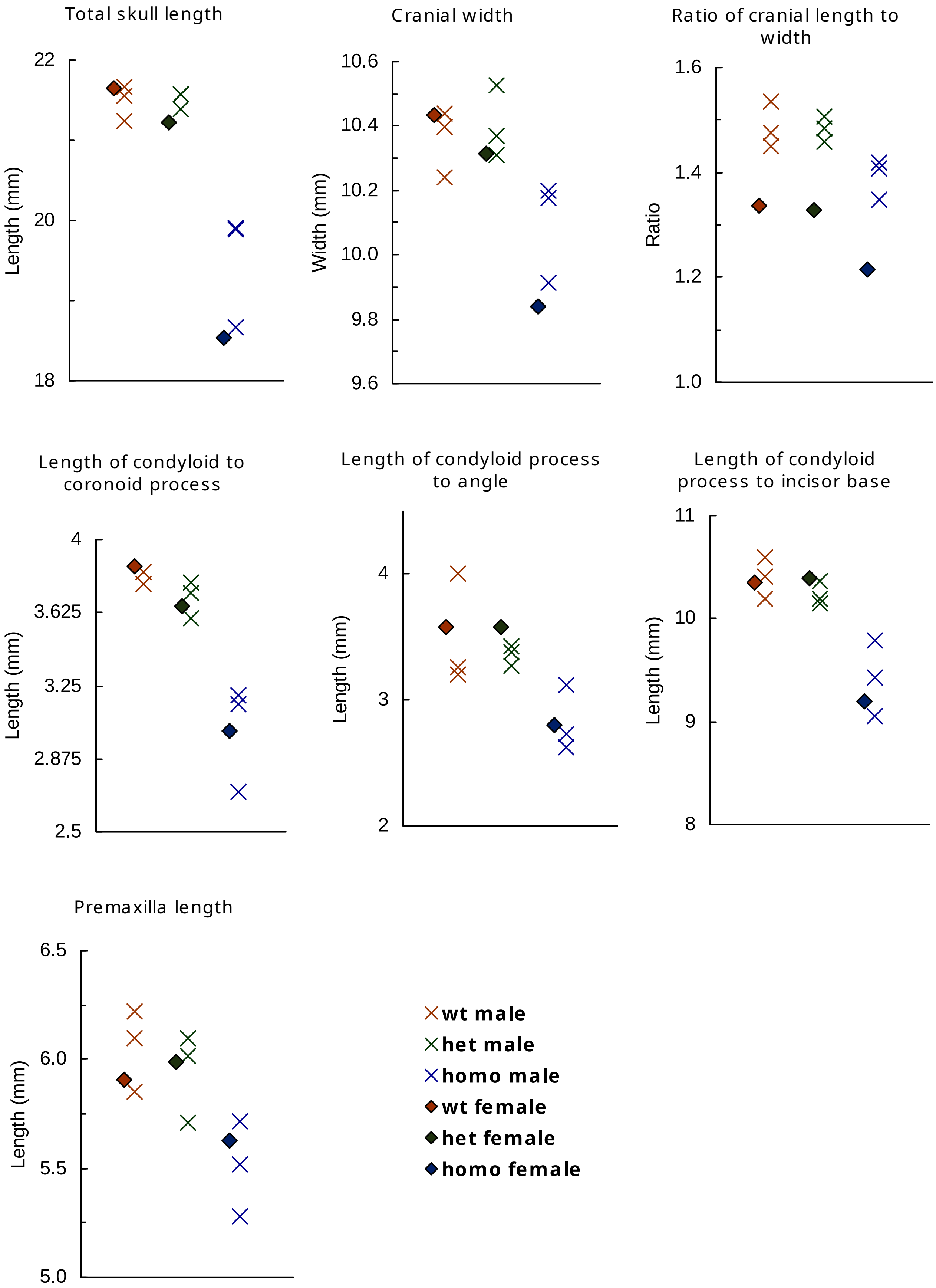
Total skull length
Length (mm)
22
20
18
Cranial width
Width (mm)
10.6
10.4
10.2
10.0
9.8
9.6
Ratio of cranial length to width
Ratio
1.6
1.4
1.2
1.0
Length of condyloid to coronoid process
Length (mm)
4
3.625
3.25
2.875
2.5
Length of condyloid process to angle
Length (mm)
4
3
2
Length of condyloid process to incisor base
Length (mm)
11
10
9
8
Premaxilla length
Length (mm)
6.5
6.0
5.5
5.0
wt male
het male
homo male
wt female
het female
homo female
